# From an Introductory Lecture on Dental Chemistry

**Published:** 1856-06

**Authors:** Geo. Watt


### FROM AN INTRODUCTORY LECTURE ON DENTAL CHEMISTRY.

BY GEO. WATT.

When material substances are properly studied, they teach lessons of great interest, and of the highest importance. Man, corporeally considered, is composed of, and surrounded by, matter. It constitutes the whole visible creation, forming a vast field of observation for finite minds. The universe and its Creator may be resolved into two grand divisions, matter and spirit; and two volumes, that of creation and that of revelation, are given us to teach that which we should know concerning them. Each volume teaches much of both divisions. While "the heavens declare the glory of God, and the firmament showeth his handiwork," we are also taught that "the worlds were framed by the word of God, so that things which are seen were not made of things which do appear."

But, as intimated in the outset, we now propose to confine our thoughts to the study of matter; and, should our minds, as we proceed, be led "through nature up to nature's God,"

it will only evidence that we are properly pursuing and profiting by our lesson.

Matter possesses both physical and chemical properties; and hence, we have two corresponding sciences, Natural Philosophy and Chemistry.

The physical properties of matter are commonly divided into general and secondary. The general are common to all bodies; the secondary exist only in some. Extension, impenetrability, mobility, indestructibility, extreme divisibility, gravitation and porosity are general properties; while softness, hardness, opacity, transparency, color, solidity, fluidity, elasticity, density, etc., are secondary.

The mechanical philosopher resolves, by virtue of these properties, to make matter act upon matter, for his own accommodation and for the benefit of his race. The hard is made to act upon the soft, the elastic upon the solid, and so on, through the whole list, till the result is the thousands of mechanical appliances which distingnish the enlightened from the savage races. The majestic steamer and the cambric needle, the railway train and the child's rattle, are alike the result of a knowledge of these principles practically applied.

The farmer, in view of the divisibility and porosity of matter, pulverizes the earth's surface, that the air and water may be freely admitted to germinate and fructify the implanted seed; and he is rewarded with pastures covered with flocks, and valleys clothed with corn.

The astronomer, using the transparency of some, and the opacity of other bodies, constructs an instrument with which he surveys the heavens, travels up the milky-way, above the clouds and beyond the sky, wanders from planet to planet, from star to star, and beholds matter, rolled up in worlds, by the plastic hand of Deity, and hurled on its revolving career.

A different view is that of the chemist. He, too, studies the properties of matter; but, strictly speaking he is not concerned with its physical properties. It is his province not to examine what bodies are, but of what they are composed. It is his privilege to discover and define the laws by which the Creator controls matter, as body is united with body, forming substances entirely new, as it were, by a sub-creation.

Let us then, for a moment, glance at the chemist's field of labor.

For want of a better mode of expression, we speak of matter being subject to various kinds of attraction; as the attraction of cohesion, which tends to reduce all substances to, and retain them in the solid state; the attraction of gravitation, or that principle which causes bodies suspended in the air to fall to the earth; and chemical attraction or affinity.

Like cohesion, affinity acts only at insensible distances; that is, substances are brought under the sphere of its influence only by apparent contact. It differs, in this respect, entirely from gravitation, which acts at all distances. It differs from cohesion, by its being exerted only between dissimilar particles, while cohesion unites similar particles. For illustration, a block of marble is composed of a vast number of particles, held together by cohesion; and each particle is as really marble as the block itself. But marble is composed of lime and carbonic acid, and these substances are altogether dissimilar, and totally different from marble. They are called the component or constituent parts of marble; and they are united and held together by affinity. The integral particles of a body are held together by cohesion, the constituent parts are united by affinity.

Every chemical phenomenon is produced by the operation of affinity; and hence, the chemical properties of bodies are dependent on its action. Its influence extends over all substances, yet it is subject to various modifications. Two substances sometimes manifest no affinity for each other; but a third substance may be added, which has an affinity for both; and a union of all three may be the result. For example, water and oil may be commingled, yet they do not unite; but if an alkali be added, a union of the three ensues, and the formation of soap is the result.

A substance often manifests unequal affinities for two other substances, and it will then quit the one for the sake of the other. A familiar example of this is afforded by alcohol, camphor and water. The first has an affinity for each of the others; but you all know that when water is added to tincture of camphor, the gum is precipitated, and floats on the surface.

The union of two substances, by affinity, is called combination; and the result is a new substance, endowed with properties peculiar to itself. When this change is accompanied by the destruction of a previously existing compound, *decomposition* is effected. A vast field is here laid open for experiment. It may be ascertained what substances will combine with each other, and in what proportions they unite, and then the properties of the new substances and their affinities must be examined and ascertained. Other substances must next be examined, to ascertain whether they are simple or compound bodies, and if compound, what are their component elements, and by what process they may be decomposed; and finally, after collecting and arranging an extended series of isolated facts, general principles may be deduced from them, and the result is *the science of chemistry.*

As the object of the chemist is to examine the relations which affinity establishes between bodies—to ascertain the nature and constitution of the compounds it produces, and to determine the laws by which its action is regulated, it follows that *Chemistrg is the science of affinity.*

This science not only embraces an extensive field, but admits of a very extended application. It justly ranks as a foundation to other sciences. The farmer, after exhausting his strength and skill, is often pained to find his fields unproductive and his crops light. He applies manure, which imparts vigor to the weeds, but none to the crops; he varies the treatment, and an abundant yield of straw, with but little grain, is the result; he changes again, and his crop has not strength to mature; again he tries, and may succeed, after losing successive crops and years of toil, while this science would at once have told him the defects of his soil, and the remedies to apply.

The physician may administer a complex prescription, and to his surprise, witnesses no results, or results totally different from those expected. An accurate chemical knowledge would allay his surprise by teaching him that he had combined incompatible substances. But I need not insist here; for this science is conceded to be an essential part of a medical education. Indeed, it seems to underlie all correct medical attainments. The botanist may classify and ascertain the general properties of plants, and may partially discover their more obvious medicinal virtues; but only the chemist can tell in what element, or compound radical, these virtues exist. He only can eliminate these hidden principles, the discovery of which has done so much to bless mankind. Only the chemist can travel through both the organic and inorganic kingdoms of nature, selecting the good and rejecting the baneful. 'Tis his province to create, as it were, new bodies, by the union of those already formed; to produce the cooling and healthful salt, from the combination of caustic poisons. 'Tis his privilege to discover and apply the antidote to the poisonous dose, whether administered by mistake, or by the hands of villainy. To him society looks for light, when dark suspicion overshadows the death of one of its members; and, by his analysis, the vilest murderer, too much of a coward even to stab in the dark, is often detected and brought to justice.

The anatomist studies the animal structure, admires the harmonious arrangements of the various organs, grows eloquent over the beautiful adaptation of each to its intended use, calls one structure bone, another, muscle, etc.; and at last, in endeavoring to penetrate still deeper into the subject, applies to chemistry, to ascertain what substances compose these various tissues; nor does he apply in vain.

The physiologist, with laudable industry, endeavors to find out the uses of the various organs; he investigates the laws of life and observes the origin, development and growth of the animal tissues; and after tracing the whole back to cell formations, his science will carry him no farther. It is then that chemistry is called in to trace these cells, the starting point of the organic kingdom, back to their original elements in the inorganic. Through his entire course, from the primary cell to the perfect animal of the highest scale, chemistry is the guide and assistant of the physiologist. And when his journey is completed, when he has, one by one, examined each secretion, she returns, and lights the pathologist through the mazes and intricacies of diseased life; ever and anon stopping to enable him to scrutinize each point of interest by the way. She tells him its condition at her late visit with his predecessor, and unfolds its present state, leaving him only, as it were, to compare results and draw conclusions.

I need not mention the influence of chemistry on the arts. You need not be told that it selects the materials for, and prepares the tools of the mechanic; that it prepares and mixes the colors for the painter; that it furnishes the materials and defines the proportions of the builder's cement; for these are plain to the most superficial observer. It is much to all of the arts—it is all to some of them.

But the question arises in your minds, "Is it any thing to Dental Science?"

Let us then, for a short time, consider its relation to, and its influence on the science of our own profession. And here you will allow me to premise, in the language of another of your professors, that "it has done more to develop and advance dental science than any other agency." Before its dawn, the nature, and of course the treatment, of the principal diseases of the dental organs were veiled in darkness and mystery. Even dental caries was supposed, by men of undoubted ability, to be produced, as that in other bones, by internal inflammation. This science teaches us that it always results from external chemical action; it detects the causes, demonstrates their action, defines their origin, suggests the remedies, and prepares them to our hand. And in the selection of materials to fill the cavities of decay, and restore the contour of the tooth, it manifests no sordid meanness; but directs us to those noble and indestructible metals, gold and platinum, discarding the whole category of alloys, amalgams, mineral pastes, etc., which none but the unprincipled, and those ignorant of the fundamental truths of this science, ever think of using.

This science teaches the dental surgeon the precise condition of the various secretions with which he is concerned, their deleterious influence when abnormal, and points out and prepares the appropriate remedial measures.

The same science selects the most suitable and only fit materials for artificial bases—prepares them for the mechanical dentist, and instructs him how to use them. It rejects the dead human, animal and ivory teeth; and, from the bowels of the earth, brings up the flinty rocks, and the metallic oxyds, and from their combinations, manufactures the pearly gem, surpassed only by the Creator's gift, the living teeth.

But it is needless to specify. A correct knowledge of chemistry will guide you safely in the paths of dental truth; while those who neglect her teachings, or spurn her advice, are left to grope their way through bogs of ignorance, prejudice and error.

Dental Surgery has taken her stand, and is entitled to rank as an honorable profession; hence, her votaries should be honorable men, and, of course, an honor to their profession. To accomplish this, they must be acquainted with the science of their profession; and this alone should be argument sufficient to induce a careful attention to chemistry. I do not mean that you must understand the whole of chemistry; for that is impossible, in our present condition. A young man of respectable talent may commence the study in his youth, and pursue it through life, yet fail to overtake it in its onward march. But does this afford a reason for neglecting it? Far from it. What would you think of the geographer who would abandon his science because he could not visit every city and district, climb each mountain, and trace each river? That science is advanced by adventurers in different fields; each pushing to the utmost, in his favorite direction. But, to pursue his subject successfully, each traveler must understand the first principles of geography.

So it is with chemistry. One applies it to agriculture; another to the mechanic arts and manufactures; another to mineralogy; another to the animal system, and so on. And, as in the former case, each must understand the elementary principles of the science, or totally fail in his effort.

While going over the laws of combination, the doctrine of affinity, nomenclature, etc., the student sometimes inquires, "What has this to do with *Dental* Chemistry?" The commercial student might with equal propriety make a similar inquiry in regard to the primary rules of arithmetic, by asking, "What has this to do with accounts?"

It will be our endeavor, then, to assist you in obtaining as rapidly as possible a correct knowledge of elementary chemistry, and in applying it to your professional wants. If we can aid you in laying a good foundation for future attainments, we shall be satisfied. I trust that I may rely on your attention and co-operation. No one can be more thoroughly aware of the difficulties that lie in our way, or more conscious of incapacity than myself; yet, relying on your zeal and industry, I expect to succeed.

And now, gentlemen, we will close these remarks. They are few; but as they are thrown together without much system, you will agree that you have enough of the sort. The ice is now broken. You have seen me, and I have looked at you. Consider yourselves acquainted, and I shall do the same. When we meet to-morrow, we will take up the subject of HEAT; and though it is sometimes considered a *dry* subject, we hope you will all become *warm* in the cause.

